# Effect of laser shock peening on microstructure and mechanical properties of laser cladding 30CrMnSiNi2A high-strength steel

**DOI:** 10.1038/s41598-023-37060-w

**Published:** 2023-06-20

**Authors:** Lingfeng Wang, Kun Yu, Xing Cheng, Tong Cao, Liucheng Zhou

**Affiliations:** 1 Science and Technology on Plasma Dynamics Laboratory, Air Force Engineering University, Xi´an, 710038 China; 2 Xi´an Aerospace Mechatronics & Intelligent Manufacturing Co., Ltd, Xi´an, 710038 China

**Keywords:** Materials science, Optical materials and structures

## Abstract

The effect of laser shock peening (LSP) on the microhardness and tensile properties of laser cladding (LC) 30CrMnSiNi2A high-strength steel was studied. After LSP, the microhardness of the cladding zone reached approximately 800 HV_0.2_, which was 25% higher than that of the substrate, while the cladding zone without LSP had an approximately 18% increase in its microhardness. Two strengthening processes were designed: groove LSP + LC + surface LSP versus LC + surface LSP. The former's tensile strength and yield strength were less than 10% weaker than those of forged materials, which is the best mechanical property recovery found in LC samples. The microstructural characteristics of the LC samples were analysed by scanning electron microscopy (SEM) and electron backscatter diffraction. Under the action of the laser-induced shock wave, the grain size of the LC sample surface was refined, the low-angle grain boundaries on the surface layer increased significantly, and the austenite grain length was reduced from 30–40 μm in the deep layer to 4–8 μm in the surface layer. In addition, LSP modulated the residual stress field, hence preventing the weakening effect of the LC process's thermal stress on the components' mechanical properties.

## Introduction

Encouraged by its outstanding features of high strength, good plasticity and toughness, 30CrMnSiNi2A high-strength steel has been widely used for critical bearing parts of mechanical components such as high-strength connectors and shafts, particularly in the aviation field (such as aircraft landing gear)^1^. Because high loads are always suddenly applied to the structural components made of 30CrMnSiNi2A high-strength steel, these parts are very susceptible to local damage and microcracks caused by wear, corrosion, and scratch, and their service life will also be seriously influenced^2^. On-site statistics show that aircraft landing gears suffer from fatigue cracks and stress corrosion cracks formed in chamfers, welds and circular holes during long-term service. The depths of these cracks go beyond their allowable range, sometimes even exceeding 5 mm, which means a recognizable fracture risk. Considering the high maintenance cost of aero components, it is necessary to study the repair technology of 30CrMnSiNi2A high-strength steel. Welding is a typical metal structure repair method in engineering. Since 30CrMnSiNi2A high-strength steel has a significant content of carbon and alloy elements that have a large liquid/solid phase interval during welding, severe segregation is commonly observed, consequently causing heat cracks and deformations^3^. Postweld quenching will form hardened martensite in the welding zone and heat-affected zone (HAZ), leading to cold cracks^4^. Therefore, this kind of high-strength steel can barely be repaired by welding and requires precise performance in the welding process, which restrains its widespread application in engineering.

Laser additive manufacturing (LAM) is considered one of the most promising repair technologies to reconstruct geometric features and restore mechanical properties for steel^5, 6^. Laser cladding (LC) is one of the LAM processes that utilize the rapid heating and cooling characteristics of laser beams to clad identical or different metal powders on the surface of components. This process has several advantages, including a dense cladding structure, high interface bonding strength, small microstructure grain size, and small component deformation^7^. Telasang et al.^8^ validated that laser cladding produces a refined microstructure that significantly improves surface microhardness. When the LAM process is applied, local damage to the 30CrMnSiNi2A high-strength steel landing gear can be repaired without disassembling, so the repair process is fast and the maintenance cycle can be significantly shortened. On the other hand, one of the disadvantages of LC is that it weakens the static strength of components^9, 10^. For instance, laser-induced heat dissipates via previously deposited materials, frequently leading to an annealing effect in the repaired zone beneath the molten pool. This may further lead to grain coarsening in the HAZ and decrease the tensile strength of the repaired material. Ning et al. validated that the microhardness and tensile properties of laser cladding high-strength steel can be improved by adjusting the laser process^11^. In addition, since the laser process has a large temperature gradient, it can easily lead to material defects, including pores and a lack of fusion. It could further produce a significant residual compressive stress field inside the cladding zone and residual tensile stress on the surface of the cladding zone^8, 12^, which seriously impacts the tensile properties of the repaired components. Thus, modulating the residual stress field and microstructure is crucial to improving the mechanical properties of LC high-strength steel components.

In current practice, heat treatment and mechanical surface treatment are standard methods to eliminate tensile stress^13–15^ and change the microstructure of the cladding zone^16^. However, in aircraft landing gear maintenance, the time cost for heat treatment is too high since component disassembly is needed. Significant attention has been given paid to surface mechanical treatment technologies due to their advantages in modulating surface stress fields and microstructures^17^. Laser shock peening (LSP) is a surface treatment technology to increase the material surface strength through laser-induced plasma impact. It can induce a residual compressive stress layer with a depth of greater than 1 mm and a surface grain refinement effect^18–20^. Morgano et al.^21^ measured the residual stress in LSPed 316L stainless steel produced by laser powder bed fusion (LPBF). The results show that the residual stress extends to approximately 1 mm below the surface. The compression effect occurs most significantly at nearly 100 μm to 300 μm from the surface. The research results of Chi et al.^22^ show that LSP transforms the residual tensile stress into compressive stress in LAM Ti17 alloy, which significantly improves the surface hardness through grain refinement and work hardening. In addition, Kalainathan et al.^23^ and Dorman et al.^24^ discovered similar conclusions using LSP with different materials. These studies show that LSP significantly reduces the tensile stress generated in the manufacturing process.

Moreover, LSP has obvious effects on microstructural changes, especially on the microstructure of martensitic laths, and thus improves the mechanical properties. Wang et al.^25^ found that the LSP-induced carbide fragments in AISI 420 martensitic stainless steel promote the nanocrystallization of martensitic laths, which contributes to an increase in strength and ductility. Their work also demonstrated that high-density dislocations and a large number of mechanical twins can be generated in the coarse α’ martensites of LAM Ti-6Al-4 V by a laser shock wave and then gradually evolve into refined α’ martensites, improving the tensile strength and ductility at the same time^26^. Iordachescu et al.^27^ studied the effect of LSP on AA2024-T351 friction stir welded joints and confirmed that LSP could improve the tensile strength and local crack resistance. Zhang et al.^24^ found that after double-side LSP, the tensile strength and yield strength of laser welded ANSI 304 stainless steel were significantly improved, and they analysed the strengthening mechanism from the perspective of elongation dimples at the fracture. LSP is considered a good choice for post-treatment to eliminate the detrimental effects produced by LAM. Component cracks may originate from internal defects or weak positions while bearing a tensile load. LSP can improve the mechanical properties of both the heat-affected zone (HAZ) and cladding zone on the LC surface but can do nothing to the treaded HAZ inside components. Lu et al.^28^ proposed an innovative laser hybrid additive manufacturing technology that combined LDED with LSP, called interlayer LSP treatment. This process can inhibit the epitaxial growth of columnar grains caused by LDED and form fine equiaxed grains between deposited layers. In addition, the residual stress field of the LDEDed specimen treated by interlayer LSP presented a gradient distribution of compressive stress in depth. The results indicated that the microhardness and tensile properties of the LDEDed specimen were significantly improved by the interlayer LSP treatment (microhardness 10%, UTS 20.8%, YS 19.6%).

Consequently, the process of LSP treatment on the groove is proposed before LC to modulate the grain size and induce high-density dislocations to improve the mechanical properties of the LC weak areas in the component. Laser cladding on a 30CrMnSiNi2A high-strength steel substrate was carried out using powder of the same material, and LSP treatments on the groove before cladding and on the surface after cladding were proposed as repair processes. The microstructural characteristics of the substrate, HAZ and cladding zone after different processes are studied by scanning electron microscopy (SEM), electron backscatter diffraction (EBSD) and transmission electron microscopy (TEM). The microhardness and tensile properties of the repaired samples are tested, and the fracture morphology characteristics are analysed. The physical mechanism of LSP to restore the mechanical properties of cladding components is discussed.

## Experimental

**Figure 1:**
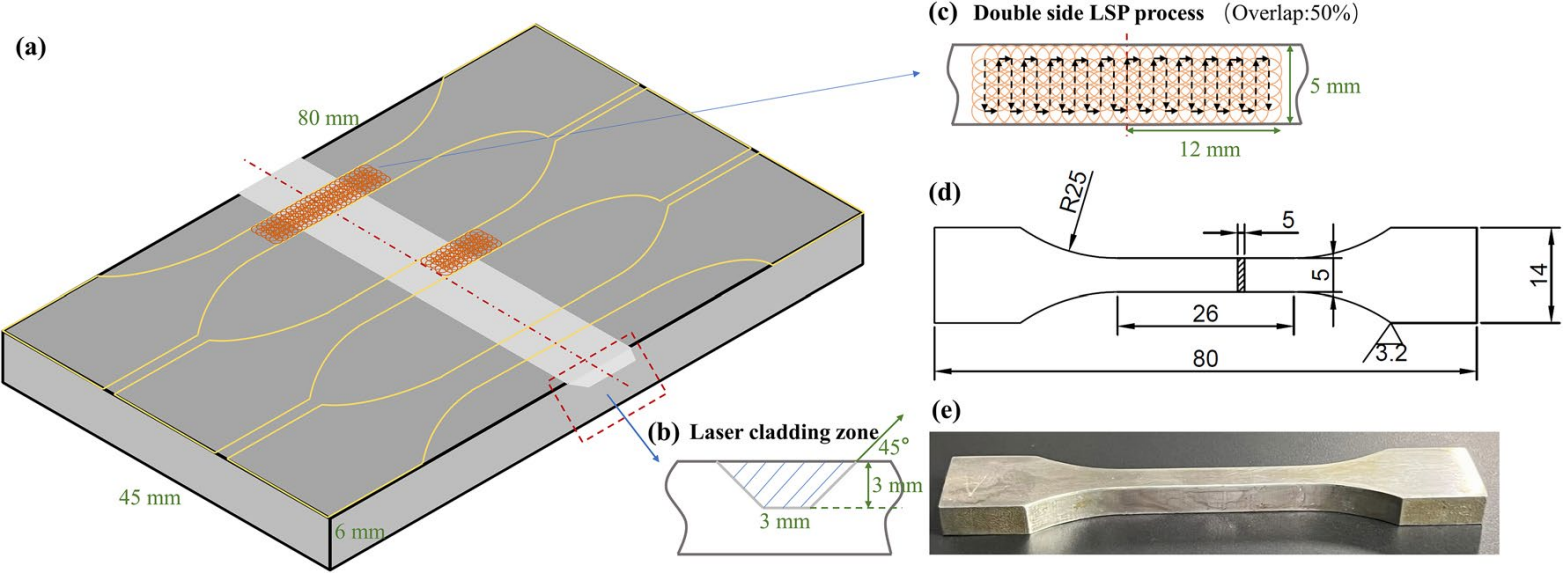
Schematics of laser cladding, laser shock peening, and sampling of mechanical property test samples. (**a**) The substrate with notch for laser cladding and sampling method of test samples; (**b**) Diagram of laser cladding; (**c**) Diagram of laser shock peening; (**d**) Designed sizes of test sample; (**e**) Physical drawing of the test sample.

The metal substrate and alloy powder used in the study are 30CrMnSiNi2A, whose chemical compositions are shown in Table 1. The substrate with a trapezoidal groove is designed for powder-feeding LC and then processed into a tensile specimen, as shown in Fig. 1. Before the LC experiment, the substrate surface was polished with SiC sandpaper, cleaned with acetone, and then dried. The alloy powder was dried in a vacuum drying oven for three hours at 85 °C.

**Table 1:** Chemical composition in wt (%) of 30CrMnSiNi2A alloy.

C	Mn	Si	S	P	Cr	Ni	Fe
0.27 ~ 0.34	1.00 ~ 1.30	0.90 ~ 1.20	≤ 0.02	≤ 0.02	0.90 ~ 1.20	1.40 ~ 1.80	Bal

An IPG YLS-2000-CT (IPG Laser GmbH, Germany) high-power laser is used in the experiment. LC is carried out in coaxial powder feeding, and the protective gas used in the cladding process is argon. The LC experiment was carried out with a laser power of 0.8 kW, a laser scanning speed of 8 mm/s, a powder feeding rate of 0.2 rpm, a laser overlap ratio of 50%, a defocus of 10 mm, and a protective gas flow rate of 15 L/min. The LSP process was performed on an Nd:YAG laser device (YD60-M165) with a wavelength of 1064 nm and a pulse width of 20 ns. The strengthening parameters of the substrate cladding groove are as follows: the laser energy is 2 J, the spot diameter is 2 mm, and the power density is 3.18 GW/cm^2^. The strengthening parameters of the surface are as follows: laser energy 3 J, spot diameter 2.2 mm, and power density 3.95 GW/cm^2^. The purpose of groove LSP is to control the microstructure of the surface layer, and it is necessary to prevent uncontrollable deformation caused by high-energy laser shock waves. The LSP after LC aims at regulating the residual stress field in the cladding zone and HAZ and introducing grain refinement, so it requires higher laser energy.

**Figure 2:**
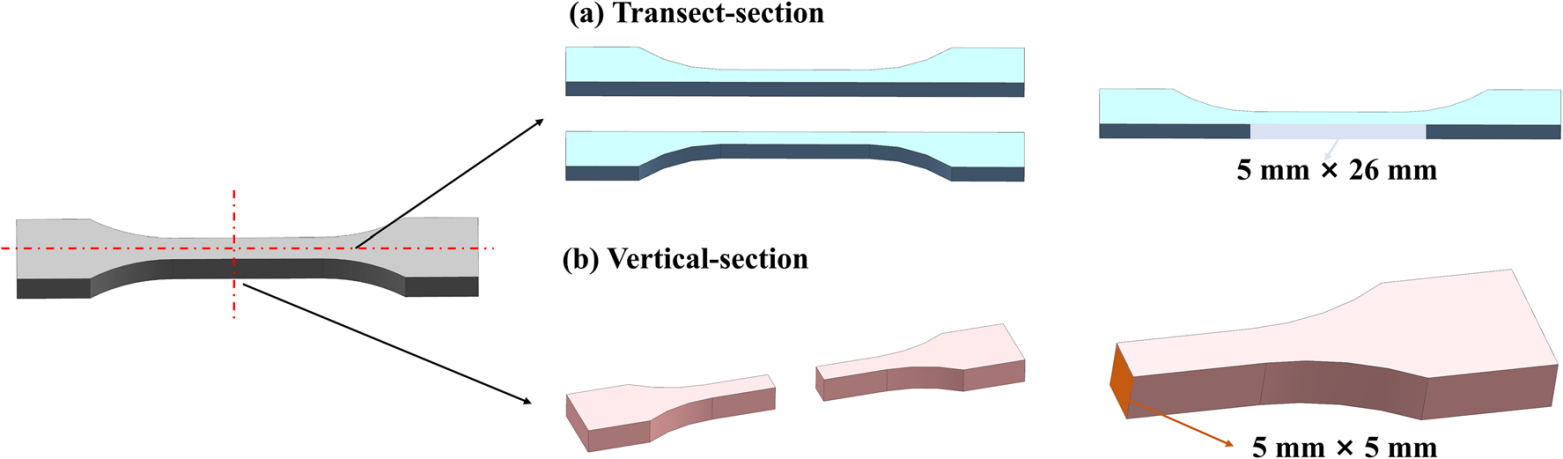
Schematic diagram of section microstructure test sampling. (**a**) Transect section; (**b**) Vertical section.

**Figure 3:**
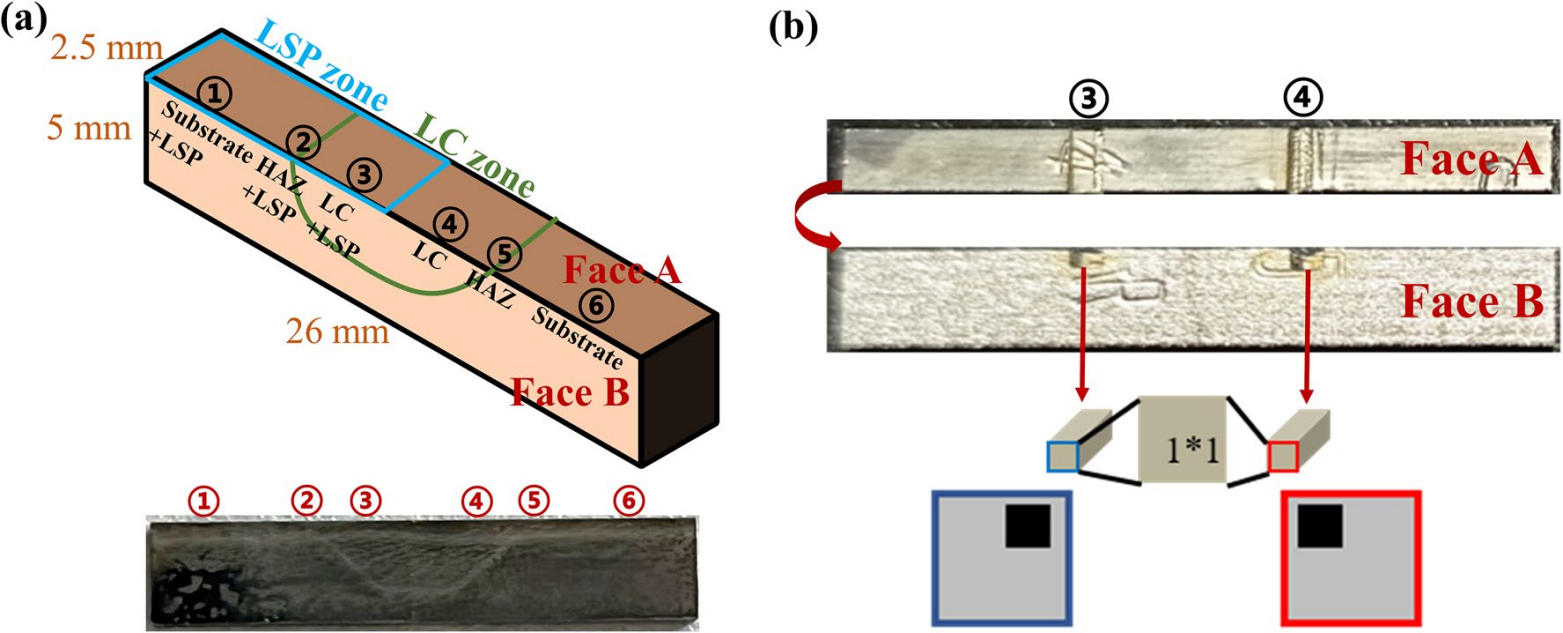
(**a**) EBSD sampling position and (**b**) TEM sampling position.

A total of 18 samples in four different states were studied, namely, matrix (3), directed energy deposition (DED, 3), LC + surface LSP (6), and groove LSP + LC + surface LSP (6). One sample of LC + surface LSP was taken for transect-section (Fig. 2a) EBSD tests (SEM: Zeiss Sigma 300, Zeiss, Germany; EBSD: Oxford Instruments, Oxford, England) after electrolytic polishing, with the sampling position shown in Fig. 3a. Slices were taken for TEM tests (TEM, JEOL, Japan) from the LC region and the ion thinned to electron transparency using a DJ2000 twin-jet electropolishing device in a polishing solution, with the sampling position shown in Fig. 3b. Another sample of groove LSP + LC + surface LSP was taken for the vertical-section (Fig. 2b) metallographic structure test (Zeiss Evo 10, Zeiss, Germany) after mechanical polishing and corrosion (in 4% nitric acid ethanol). The rest of the samples were used for mechanical property tests. The microhardness tests were carried out on an HV-1000A Vickers indenter (HUAYIN, China) for samples of different states, with a load of 200 gf and a dwell time of 15 seconds. The indentation spacing at the same position was 0.5 mm, and the average value was taken after three tests. The tensile properties were tested on an MTS Exceed E45.305 electronic universal testing machine (MTS, America).

## Results and discussion

### Microstructures

**Figure 4:**
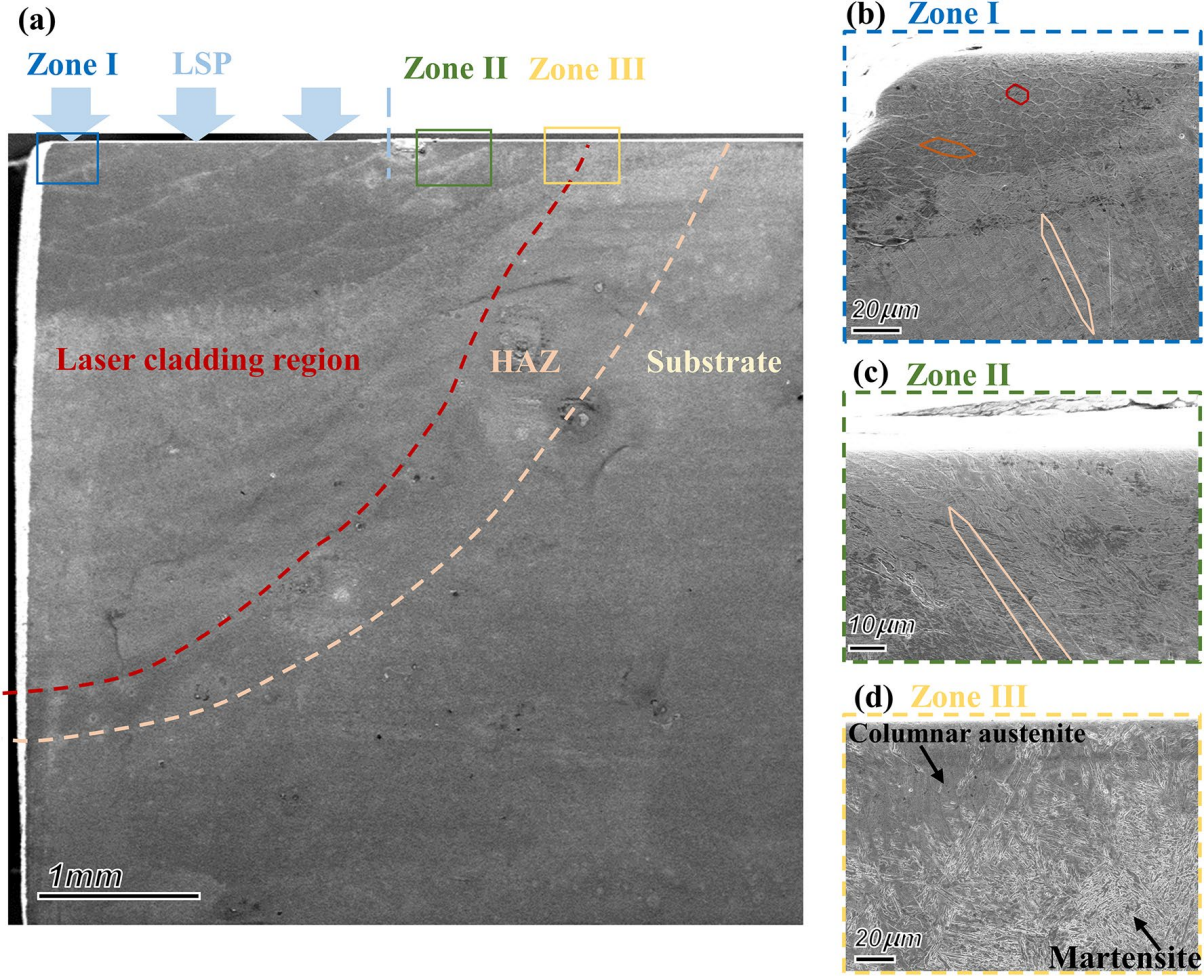
SEM of laser cladding sample transect-section. (**a**) The global image; (**b**) Local enlarged image of the LSPed cladding zone (Zone I in the global image); (**c**) Local enlarged image of the untreated cladding zone (Zone II in the global image); (**d**) Local enlarged image of the combination of the LC region and HAZ (Zone III in the global image).

Detailed observation is carried out on the transect section samples, including the LSPed cladding zone, untreated cladding zone, HAZ and substrate, as shown in Fig. 4. It can be seen from the global image (Fig. 4a) that the LC region is divided into upper and lower regions: the upper region presents a dark structure separated by light thin layers (mainly columnar austenite crystals), while the lower region is a uniform light structure (mainly lath martensite crystals). The multilayer multichannel LC process involves thermal cycling of fast heating and cooling. In the process, the ferrite in the metal powder would be austenitized under heating and then transformed into martensite under fast cooling (the martensite transformation temperature of 30CrMnSiNi2A high strength steel is approximately M_s_ = 300 ℃^29^). The lower layer of the LC region is first affected by laser heat, so it transforms fully from austenite to martensite and forms a uniform martensite structure. The fast heating and cooling function of the LC process is similar to that of the annealing process, resulting in a hardness decrease, refined grain and microstructure defects improving while austenite transforms into martensite. With the increase of the LC layers, the heat effect accumulates gradually. The temperature of the material increases, and the cooling speed slows down. All these factors result in the transformation of austenite to martensite occurring only on the surface of the cladding layer in contact with the protective air, while other cladding structures retain austenite. In addition, Cr, Ni, and other elements dissolved into austenite under high temperatures prevent the transformation of austenite to martensite when the temperature decreases; thus, the materials in the region approximately 1 mm from the surface layer present an austenite structure separated by martensite.

To further study the LSP effect on the microstructure of the LC sample surface layer, Zone I is enlarged for detailed observation, and a significant grain refinement effect is found (see Fig. 4b). The material structure below 60 μm from the surface layer presents long columnar austenite crystals growing along the thickness direction with a grain length of approximately 30-40 μm. Grain refinement appears in the columnar austenite crystals under the effect of LSP as it approaches the surface layer. The results are as follows: (a) short columnar austenite crystals form within the range of 30-60 μm from the surface layer with a grain length of approximately 15-20 μm, and (b) equiaxed austenite crystals form within 0-30 μm from the surface layer with a grain size of nearly 4-8 μm. This phenomenon is wholly attributed to the shock wave effect induced by LSP. Fig. 4c shows the surface structure of the untreated LC region. Long columnar austenite crystals can be seen in the figure with a grain length of 30-40 μm, consistent with the deep crystal structure of the LC region after LSP. The austenite grain size in the untreated LC region is larger than that in the LSPed cladding zone, and the shape is not equiaxed. This explains the lower surface microhardness of the untreated LC region compared to the LSPed cladding zone. The phase compositions of both the LSPed region and untreated region are almost identical, implying that the LSP process does not cause phase transformation in the surface layer. Fig. 4d shows the surface layer structure at the junction of the untreated LC region and the HAZ. It shows columnar austenite crystals with transformed martensite crystals in the LC region and complete acicular martensite crystals in the HAZ.

**Figure 5:**
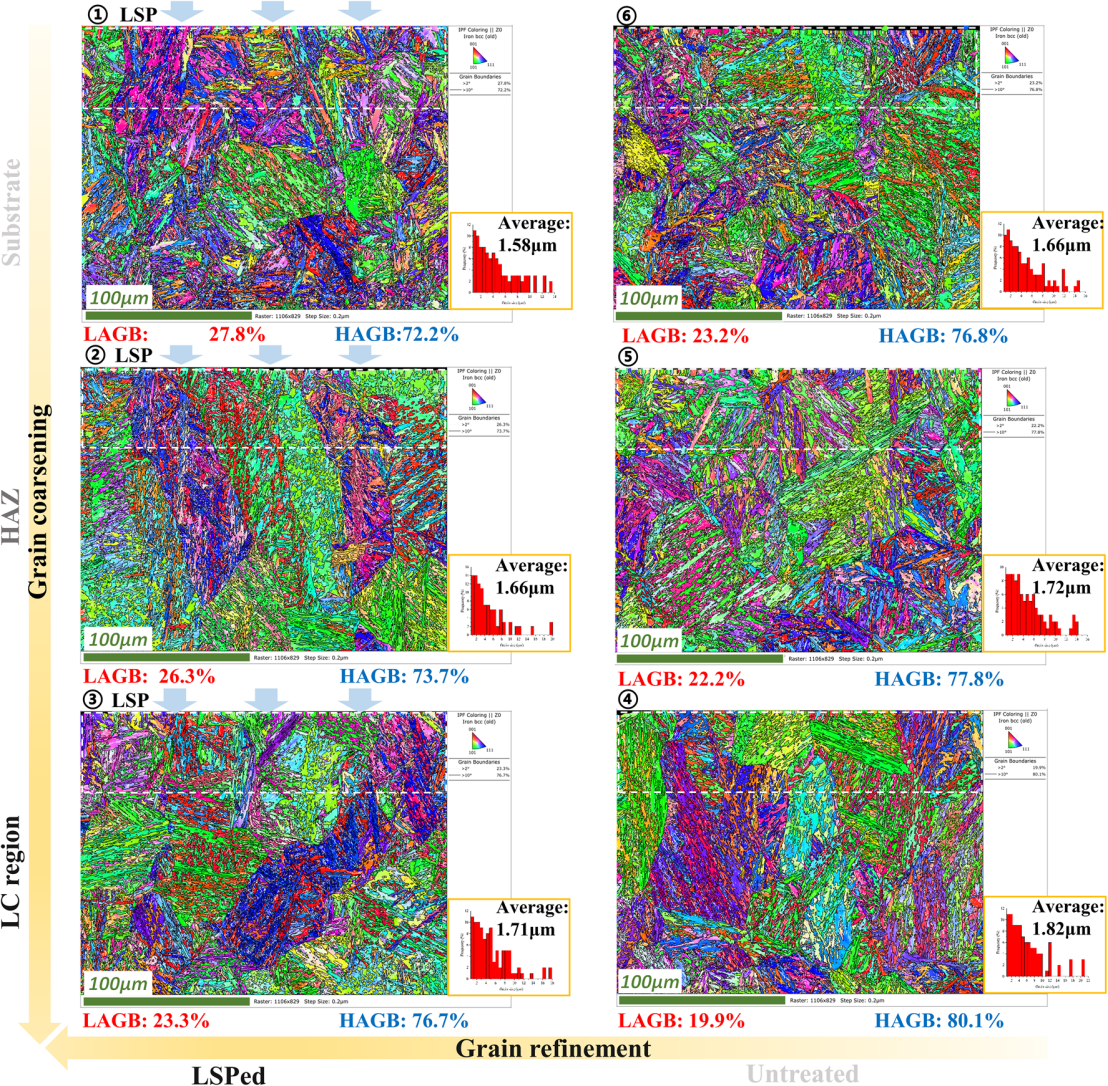
EBSD of laser cladding sample transect-section.

EBSD and TEM tests are then carried out to characterize the crystallographic characteristics of the materials, and the analysis is focused on the effect of LSP on the surface structure. Fig. 5 shows the transect-sectional EBSD morphologies of the surface layer at six regions of the LC + surface LSP sample. The grain boundary (G.B.) maps in Fig. 5 show the distribution of two types of grain boundaries, i.e., LAGBs (low-angle grain boundaries with a grain misorientation angle between 2° and 10°) and HAGBs (high-angle grain boundaries with a grain misorientation angle above 10°). LAGBs occupied a higher volume fraction at all regions on the LSPed sample compared with the untreated sample. It is well known that dense dislocations contribute to forming small subgrains with LAGBs^30, 31^. EBSD results validated that the LSP process induced high-density dislocations and promoted grain refinement in the substrate, HAZ, and cladding zone, thus effectively modulating the microstructure. More detailed analysis is carried out in subsequent TEM tests, which explains the improvement in the mechanical properties of the LC 30CrMnSiNi2A sample after LSP. In addition, Aztec Crystal software is used to statistically calculate the grain dimensions based on EBSD test data and to analyse the dimensional differences of surface grains in different regions, as shown in Fig. 5. The average (martensite) grain dimensions at the surface layer within 60 μm in regions ① to ⑥ are 1.58 μm, 1.66 μm, 1.71 μm, 1.82 μm, 1.72 μm and 1.66 μm, respectively. Obvious grain refinement could be observed in the LSPed region compared to untreated regions, while in the substrate, HAZ and LC zones, it shows a tendency of gradual coarsening of grains. This reflects that the LC process makes the martensite grain coarsen, but the LSP induces obvious grain refinement in the surface layer.

**Figure 6:**
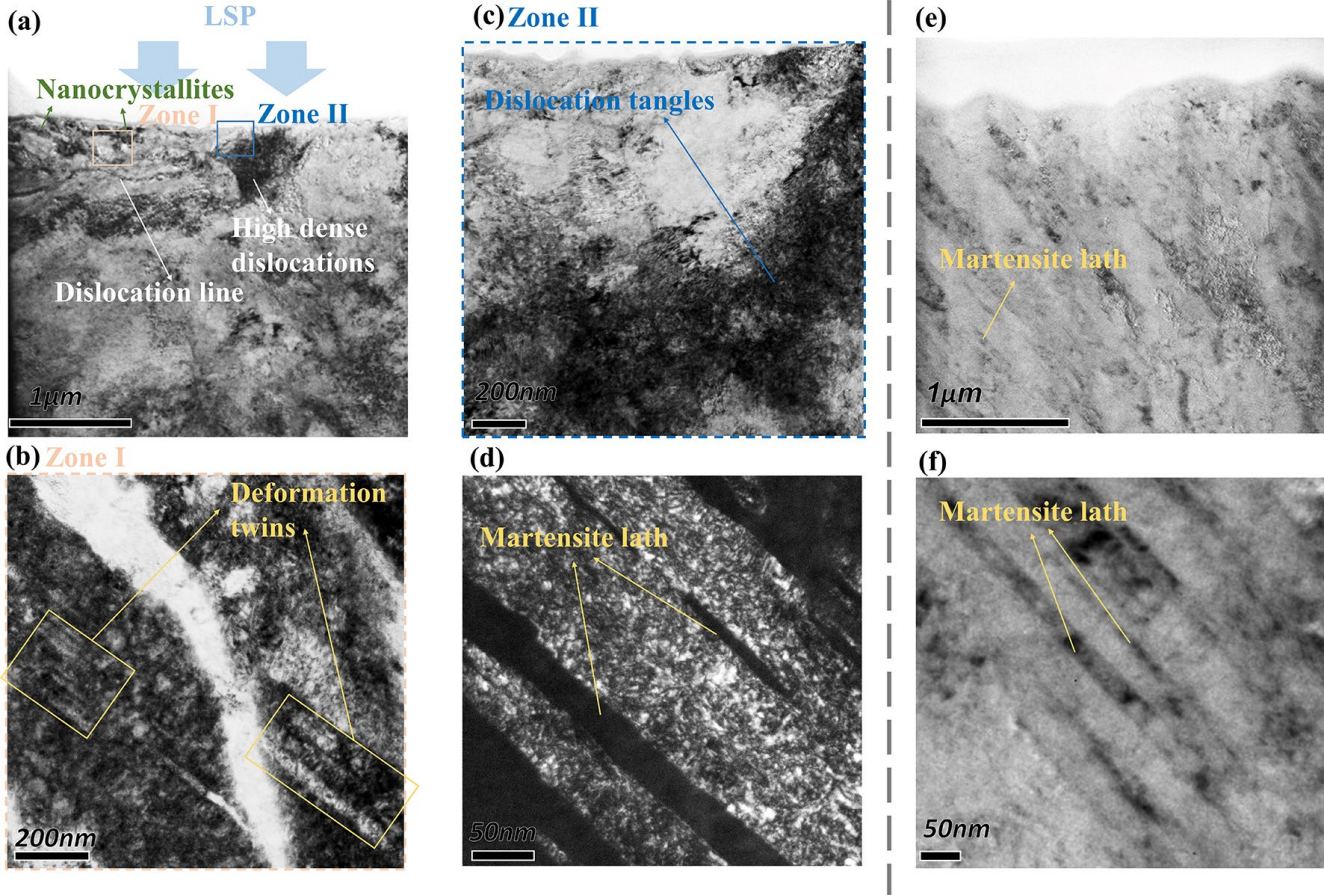
TEM of laser cladding sample transect-section. (**a**) Global image of the LSPed region; (**b**) high-magnification image of deformation twins; (**c**) high-magnification image of dislocation tangles; (**d**) martensite laths in the LSPed region; (**e**) global image of the untreated region; (**f**) martensite laths in the untreated region.

The microstructural features of the surface layer are further characterized by TEM observations. Figure 6 presents typical TEM images at a depth of 5 μm to the topmost surface of the LC region, in which (a) to (d) are LSPed regions (numbered ③ in Fig. 3), and (e) to (f) are untreated regions (numbered ④ in Fig. 3). Typical deform-induced microstructures such as dislocation lines and dislocation tangles can be observed in the LSPed surface region, as shown in Fig. 6a. High-density dislocations appear at a depth of 1 μm to the topmost surface (Fig. 6c), with uneven distribution. It is mentioned in the literature^32, 33^ that such dislocation tangles could transform into subgrain boundaries, and during the course, the original coarse grains are separated into subgrains. Compared with the untreated LC region (Fig. 6e), the density of dislocation structures in grains of the LSPed region surface layer increases greatly, and fine nanocrystalline can be observed. In addition, the TEM image of the surface layer also shows some deformation twins with a width below 50 nm, as evident in Fig. 6b, which implies the activity of deformation twins under the action of the laser shock wave. It is well known that deformation twins are activated to accommodate the deformation when metal material undergoes severe plastic deformation and dislocation sliding is unable to match the applied strain rate^34^. A small amount of fine martensitic laths can be observed in the LC region (Fig. 6d and f). This is due to the remarkable heat accumulation effect of layer-by-layer cladding in later processing: the cladding layer cools down slowly and leads to an incomplete transformation of austenite to martensite. A large amount of austenite remains in this region, while only a small amount of martensite is formed.

## Mechanical properties

### Microhardness

**Figure 7:**
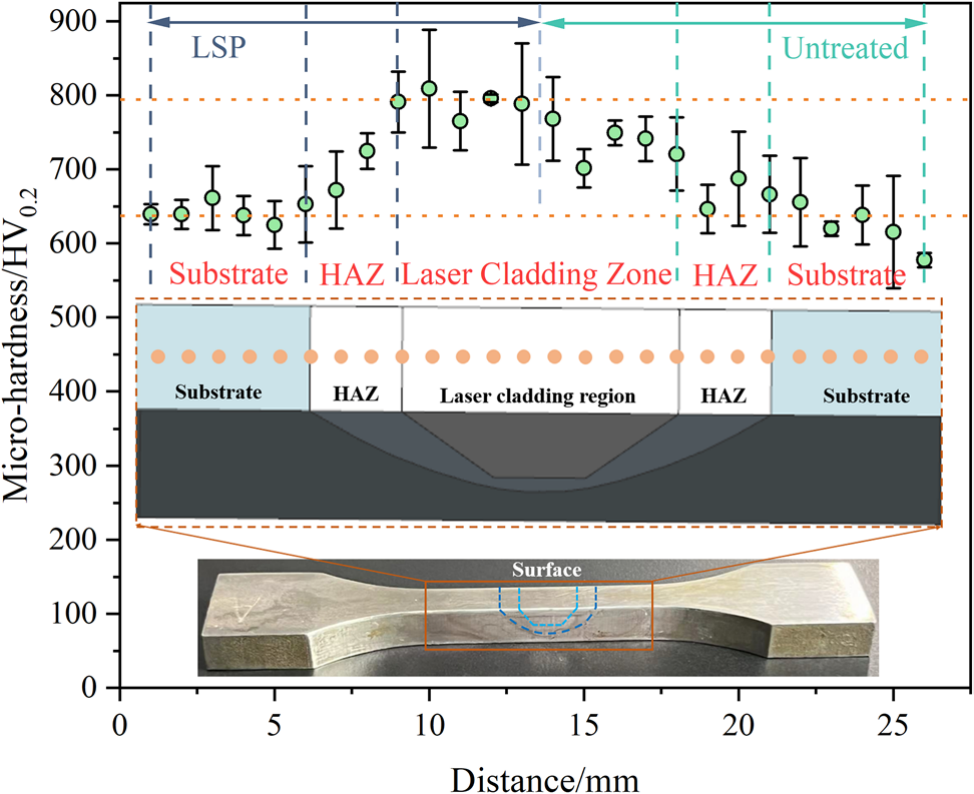
Surface microhardness of different regions on laser cladding samples with and without LSP treatment. (OriginPro 2022 (64-bit) SR1 v9.9.0.225 https://www.originlab.com/index.aspx?go=Support&pid=4440).

Figure 7 shows the microhardness distribution on the surface of the LC 30CrMnSiNi2A sample after LSP. The six positions tested in turn are the LSPed substrate, LSPed HAZ, LSPed cladding zone, untreated cladding zone, untreated HAZ, and untreated substrate. The hardness increases gradually from the substrate and HAZ to the cladding zone and shows consistency in both LSPed and untreated surfaces. The hardness of the cladding zone increased by 25% after LSP compared with that of the substrate, while the hardness of the untreated surface increased by approximately 18%. This is due to the heat accumulation effect of the LC process. The material near the surface layer experienced a higher temperature and a slower cooling rate, which inhibited the transformation of austenite to martensite.

In general, LSP treatment significantly improved the surface microhardness of 30CrMnSiNi2A high-strength steel, which is consistent with previous research^20, 35^. The surface microhardness increase after LSP can be attributed to the dense dislocations and deformation twins. Under the coupling action of an ultrahigh strain rate and high temperature, dislocations with high density are introduced into the material, resulting in dislocation strengthening^30^. This effect appears not only in the cladding zone but also in the substrate and HAZ, which has been verified in many laser welding works^16, 36^.

**Figure 8:**
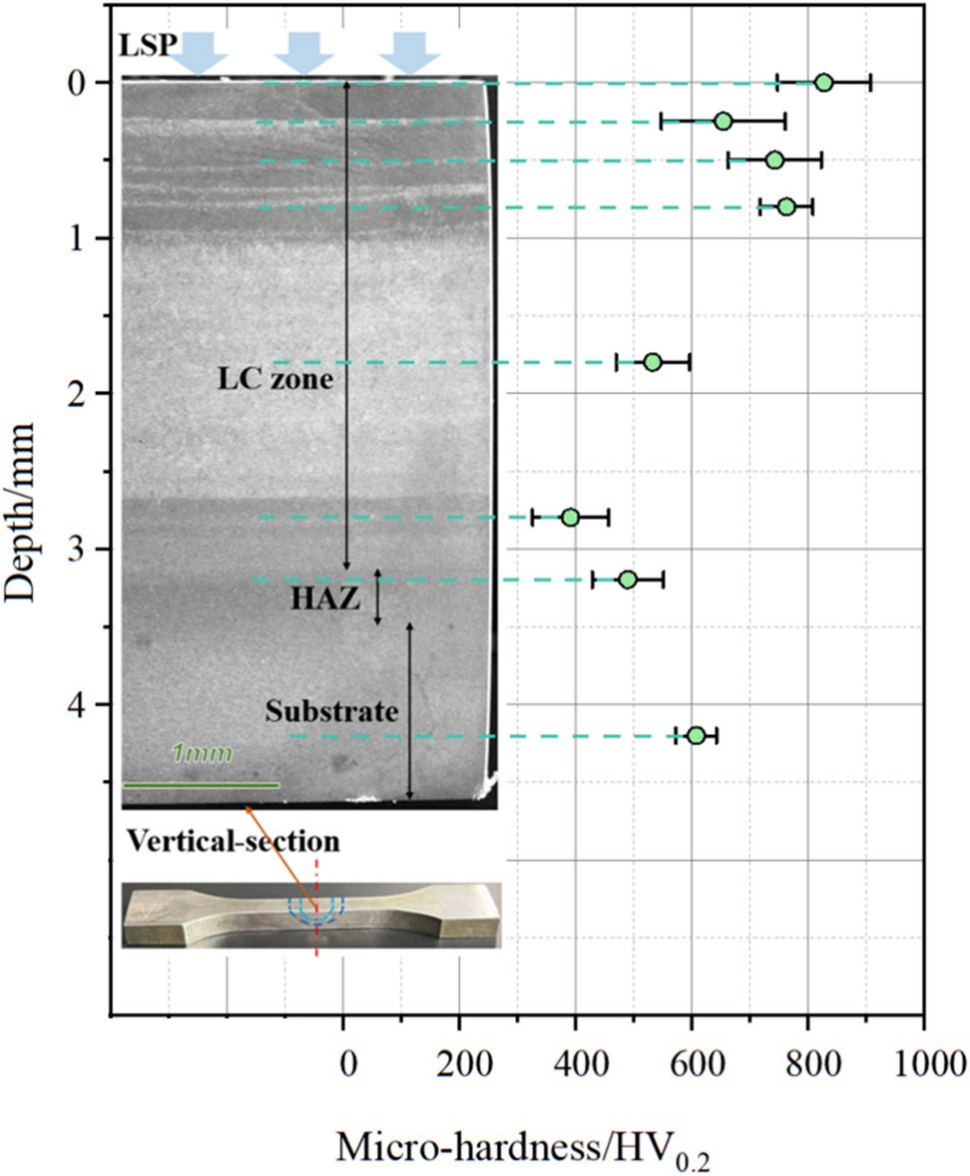
Vertical-section microhardness of different regions on the laser cladding sample with LSP treatment. (OriginPro 2022 (64-bit) SR1 v9.9.0.225 https://www.originlab.com/index.aspx?go=Support&pid=4440).

Figure 8 shows the vertical-sectional microhardness distribution of the 30CrMnSiNi2A laser cladding sample after LSP. The test results of the substrate are primarily consistent with the surface in hardness, which is approximately 600 HV_0.2_. The hardness of the HAZ and cladding zone beyond 1 mm depth is lower (nearly 500 HV_0.2_), and the martensite phase dominates these two zones. The cladding zone within 1 mm depth has a higher hardness, but it can be divided into two parts (see Fig. 4a): the black part is a columnar austenite crystal with higher hardness, while the white part is a long acicular high carbon martensite crystal with coarser grains and lower hardness. The surface layer treated with LSP has the highest hardness exceeding 800 HV_0.2_, which is consistent with the results of the surface microhardness test. SEM analysis (Fig. 4b and c) shows that the grains in the surface layer of the sample are refined after LSP, and the columnar/long axial austenite crystals are compressed into flat equiaxed austenite crystals, mainly contributing to the improvement in surface microhardness after LSP.

### Tensile properties

**Figure 9:**
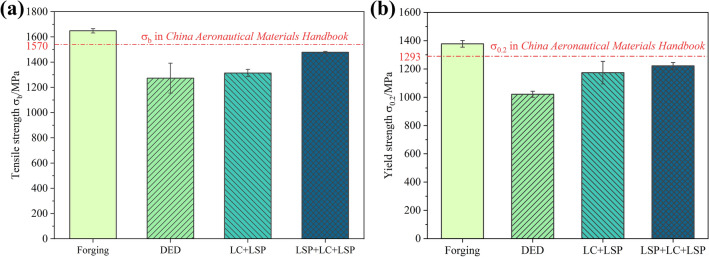
(**a**) Tensile strength and (**b**) yield strength. (OriginPro 2022 (64-bit) SR1 v9.9.0.225 https://www.originlab.com/index.aspx?go=Support&pid=4440).

Figure 9 shows the tensile properties of the 30CrMnSiNi2A high-strength steel samples in four states. Compared with the data from the *China Aviation Materials Handbook*, the mechanical properties of DEDed (Direct Energy Deposition) 30CrMnSiNi2A high-strength steel are the worst. The tensile strength and yield strength decrease significantly to 77.2% and 74.1% of the substrate, respectively. The mechanical properties of laser cladding 30CrMnSiNi2A samples after LSP improved, especially in yield strength. It is worth mentioning that the mechanical properties of the groove pre-LSPed samples (groove LSP + LC + surface LSP) improved more significantly, and both tensile strength and yield strength reached a high level that was only slightly lower (< 10%) than those of forged materials. This shows that the groove pre-LSP process can effectively restore the tensile properties of LC 30CrMnSiNi2A high-strength steel.

### Fractography

**Figure 10:**
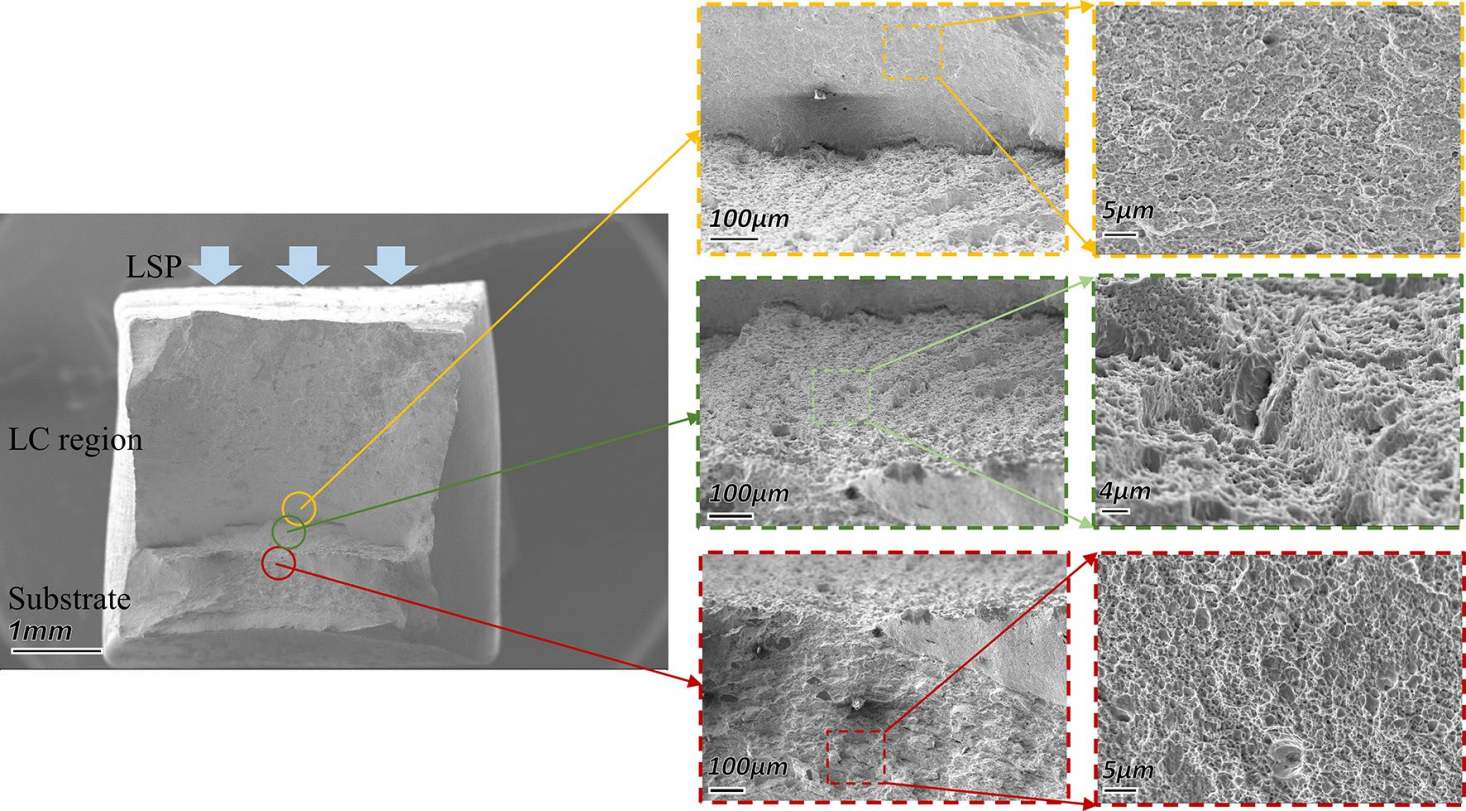
Tensile fracture of the LC + surface LSP sample.

**Figure 11:**
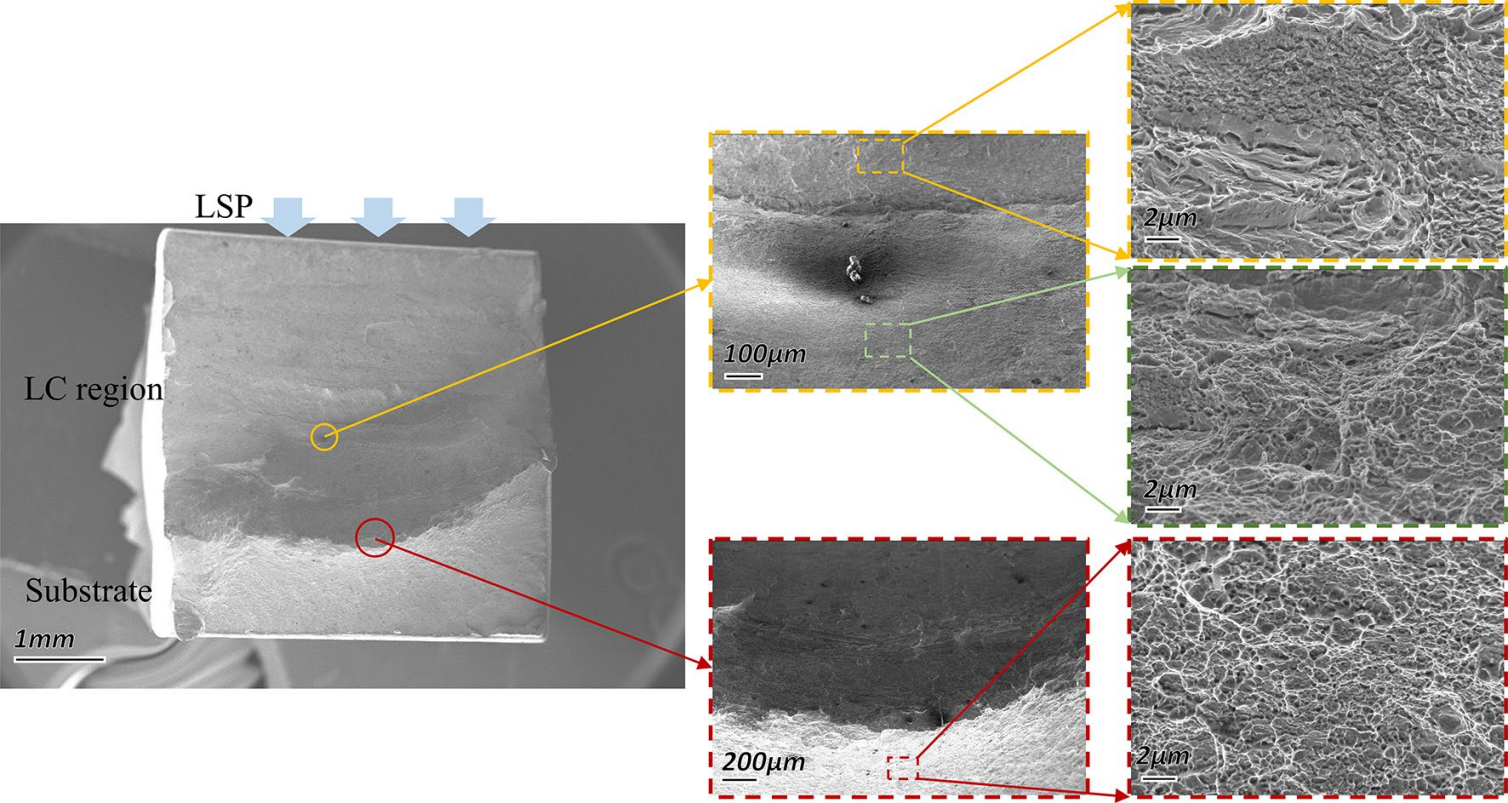
Tensile fracture of the groove LSP + LC + surface LSP sample.

Figures 10 and 11 show the tensile fractography of the LC + surface LSP sample and the groove LSP + LC + surface LSP sample. The dimples in the cladding zone and the substrate of the two samples show similar characteristics. Dimples in the sample substrate are large and deep, showing high plastic toughness and high tensile strength. The dimples in the cladding zone are small and shallow, with low plasticity and low tensile strength. Therefore, cracks first appear in the cladding zone of the two samples under tensile load and then extend to the HAZ.

The HAZ and substrate in the LC samples are mainly lath martensite, which reduces the stress concentration at the crack propagation front and improves the plasticity of the material. At the same time, martensite and columnar austenite growing perpendicular to the load direction in the cladding zone prolong the crack propagation path and make its elongation greater than that of the base.

When the crack extends to the initial position of the cladding zone, the fracture characteristics of the two types of samples are different. Since the coarse-grained martensite in the substrate of the LC + surface LSP samples could not combine tightly with the fine-grained martensite transformed from austenite during the LC process, the fracture on the LC + surface LSP samples is porous, providing conditions for rapid crack propagation. Fortunately, the large temperature gradient formed between the remelting pool and the substrate under the laser effect at the beginning of the cladding process introduces thermal stress into the groove surface and reduces the load threshold which may promote crack propagation. On the other hand, the fracture on the groove pre-LSPed LC samples presents a dense surface, and the size and depth of the dimples are similar to those of the substrate. The LSP performed on the groove introduces a residual compressive stress field that counteracts the tensile stress caused by the thermal effect during the LC process and greatly reduces the influence of thermal stress on crack growth^37^. Moreover, the combination between the substrate and the cladding zone is improved since the martensite grain on the groove surface is refined by the laser-induced shock wave, which consequently increases the resistance to crack propagation. Therefore, the tensile properties of the groove LSP + LC +surface LSP samples are higher than those of the LC +surface LSP samples.

## Conclusions

To summarize, this study demonstrated the recovering effect of LSP treatment on the mechanical properties of LC 30CrMnSiNi2A high-strength steel samples and explained the mechanism of microhardness and tensile property improvement from the perspective of microstructure and stress modulation. The differences in tensile fractures between the two LSP processes were analysed. Conclusions drawn from the work are as follows:

1. The surface microhardness of the 30CrMnSiNi2A high-strength steel specimen was significantly increased by LSP to nearly 800 HV_0.2_, which is 25% higher than that of the untreated substrate (approximately 600 HV_0.2_). The microhardness at the cross-section within 1 mm depth to the surface is higher than that of the substrate, while the microhardness of the cladding zone with a depth of more than 1 mm to the surface is lower than that of the substrate (approximately 500 HV_0.2_), which could be attributed to the phase transformation caused by the thermal accumulation effect of the multilayer and multichannel LC process.
2. The laser-induced shock wave has a significant grain refinement effect on the LC sample's surface layer during the LSP process; thus, LAGBs increased obviously. A large number of dislocations are induced, deformation twins are formed, and the original coarse grains are refined. Nanocrystals can be observed in the surface layer. The surface structure of the untreated LC sample is columnar austenite with a grain length of 30-40 μm, similar to the grain size of the deep layer (deeper than 60 μm) of the LC + surface LSP sample. On the surface layer of the LSPed sample, the columnar austenite crystal is transformed into an equiaxed austenite crystal under the action of the laser-induced shock wave, with a grain size of only 4-8 μm.
3. Compared to the DED process, both the LC + surface LSP and groove LSP + LC + surface LSP treatments can restore the tensile properties of 30CrMnSiNi2A high-strength steel, during which the effect of the groove LSP + LC + surface LSP process is more significant with tensile strength and yield strength only 10% lower than forged materials.
4. The tensile fractography in the cladding initiation region of the LC + surface LSP samples is porous, while that of the groove LSP + LC + surface LSP samples is not. Similar to the substrate, the fracture in the cladding initiation region is flat with large and deep dimples, implying higher plastic toughness and tensile strength.

## Data Availability

The datasets used and/or analysed during the current study are available from the corresponding author on reasonable request.
